# Intravenous lidocaine versus dexamethasone to prevent postoperative vomiting in children tonsillectomy: a prospective randomized controlled trial

**DOI:** 10.11604/pamj.2022.42.190.32171

**Published:** 2022-07-08

**Authors:** Imen Zouche, Ayoub Ben Salem, Salma Ketata, Mariem Keskes, Abdelhamid Karoui

**Affiliations:** 1 Department of Anesthesiology, Habib Bourguiba University Hospital, Sfax, Tunisia,; 2 Faculty of Medicine, University of Sfax, Sfax, Tunisia

**Keywords:** Tonsillectomy, children, oral intake, vomiting, dexamethasone

## Abstract

**Introduction:**

we evaluate the effectiveness of lidocaine infusion compared to the intravenous dexamethasone and placebo on postoperative vomiting and first oral intake in children post tonsillectomy.

**Methods:**

we conducted a prospective double-blinded randomized and controlled clinical trial involving children aged between 3 and 13 years proposed for elective tonsillectomy without or with adenoidectomy under general anesthesia. They were randomized into 3 groups: lidocaine group included patients who received intravenous bolus of 2 mg/kg lidocaine over 5 minutes after the induction of anesthesia. Then, they received an infusion of 1.5 mg/kg/h until the end of the surgical procedure, dexamethasone group included patients who received intravenous dexamethasone 0.15 mg/kg administrated over 5 minutes after the induction of anesthesia followed by an identical rate of 0.9% saline and the saline group included patients who received an equivalent volume of 0.9% saline. For data analysis, we tested the normality of variables using the Shapiro-Wilk test. We used analysis of variance (ANOVA) or the Kruskal-Wallis test for between-group comparisons, as appropriate. The X^2^ test and Fisher´s exact test were used for inferences on proportions. A two-sided P-value was considered significant when p≤0.05. All analyses were performed with IBM SPSS≤ 25.0.0 for windows.

**Results:**

eighty-three (83) children were analyzed in our study and randomized into 3 groups: 27 children for each lidocaine and dexamethasone group and 29 children for the Saline group. The demographic data were not significantly different between the three groups such as age (p=0.246), gender (p=0.378), and body mass index (BMI) with p=0.233. The duration of surgery and anesthesia was also comparable in the 3 groups (p=0.893). Patients of the lidocaine and dexamethasone group had at least one episode of retching, vomiting, or both less than the saline group in the post-anesthetic care unit with p respectively 0.015 and 0,035, and in the ward with p respectively 0.004 and 0,038 Without a significant difference between the dexamethasone and the lidocaine group. The time to the first oral intake was significantly shorter in the dexamethasone group and the lidocaine group compared with the saline group (p=0.0001) with no statistical difference between the dexamethasone and lidocaine groups.

**Conclusion:**

lidocaine infusion is as effective as intravenous dexamethasone on postoperative vomiting as well as on oral intake in children post tonsillectomy.

## Introduction

Tonsillectomy, without or with the removal of the adenoids, is a very, common worldwide procedure performed on children [1]. Postoperative vomiting (POV) very frequent complication of tonsillectomy, with an incidence ranging from 23% to 73% when children don´t take anti-emetic prophylaxis [1]. It might lead to dehydration because of the decrease in oral intake, and thereby it increases the risk of early hospital readmission [2]. Several pharmacological drugs have been studied to prevent POV. Many of them are available but costly or have unpleasant side effects such as bleeding, extrapyramidal symptoms, and cardiac rhythm disturbances, which reduce their cost-effectiveness [3]. Intravenous corticosteroids such as dexamethasone were shown to reduce vomiting after tonsillectomy and improve postoperative analgesia and oral intake for 24 hours [4-6]. Very few studies have focused on the infusion of lidocaine in children. Only one study was published, it didn´t show any advantage in children undergoing tonsillectomies and was underpowered [7]. The objective of this study was to evaluate the effectiveness of the infusion of lidocaine compared to intravenous dexamethasone on vomiting and oral intake in children post-tonsillectomy.

## Methods

**Study design and setting:** we conducted a prospective double-blinded randomized and controlled clinical trial involving children proposed for elective tonsillectomy under general anesthesia.

**Study population:** before surgery, children who fulfill entry criteria of the trial were selected by an anesthesiologist not involved in the clinical care. Inclusion criteria were children aged between 3 and 13 years, American Society of Anesthesiologists (ASA) I or II, and scheduled for elective tonsillectomy with or without adenoidectomy under general anesthesia. We didn´t include children with a history of congenital or acquired bleeding disorders, asthma, intellectual impairment, history of recent inflammation of the upper airways, obesity, diabetes mellitus, known congenital conduction, or cardiac rhythm disorders, gastroesophageal reflux, hista levelliver or renal insufficiency, seizures. Children known to be allergic and who received psychoactive, anti-emetic, analgesic, or antihistamine drugs within the 72 hours before the surgery were not included as well. We excluded from the study, patients who have missed data or non respect of the protocol of the study. For the sample size, without POV prophylaxis, more than 70% of children had at least one episode of vomiting in the postoperative period [8]. Based on this, we needed 25 patients per group to test a difference in proportions of 50% with a power of 80% and a level of 0.05. We enrolled 86 subjects to allow potential dropouts.

**Study randomization and allocation:** subjects were randomized according to a sequence generated by the website: (sealed envelope|randomisation and online databases for clinical trials into 3 groups). Lidocaine group was assessed to receive a bolus of 2 mg/kg of intravenous lidocaine over 5 minutes then they received an infusion of 1.5 mg/kg/h until the end of the surgery, dexamethasone group was assessed to receive intravenous dexamethasone 0.15 mg/kg bolus administrated over 5 minutes after the induction of anesthesia followed by an identical rate of 0.9% saline. The saline group was assessed to receive an equivalent volume of 0.9% saline. To maintain the blindness of the study, an anesthesiologist, who was not involved in the anesthesia care, prepared lidocaine, dexamethasone, and 0.9% saline in 20 ml syringes based on the randomization list. During the whole study period, an anesthesiologist performing the intraoperative and postoperative evaluation, medical staff who take care of children (nurse, anesthetist, and surgeon), and parents were not aware of the group allocation.

### Interventions

**General anesthesia:** all children were unpremeditated and fasted for at least 6 hours. Before anesthesia, standard monitoring was applied (electrocardiogram, pulse oximetry, and noninvasive arterial blood pressure) then 3 minutes of oxygenation was performed and 8% inspired sevoflurane in a mixture of air 50% and oxygen 50% by face mask was given to all children until the eyelash reflex had disappeared. After that, intravenous access was attained, propofol (4 mg/kg) and suxamethonium (1 mg/kg) were administrated, and endotracheal intubation was performed, anesthesia was subsequently maintained with sevoflurane 1-3% in a mixture of air 50% and oxygen 50% and alfentanil (30 µg/kg). The sevoflurane concentration was adjusted to keep the heart rate and blood pressure within 25% of pre-induction values. All children received 30 ml/kg of a solution of Ringer´s lactate® over the surgery. After induction, subjects were randomly allocated, using a table of computer-generated random numbers, to receive one of the three solutions over the surgery period. All tonsillectomies were performed in the same surgery technique by dissection of the pericapsular plane. The surgeon used a cold steel technique, which is the common practice in our hospital. Adenoidectomy was performed with adenectomy, and hemostasis was obtained with gauze packing. The surgical techniques used in tonsillectomies and adenoidectomies were standardized for all cases. All children were given intravenous acetaminophen (15 mg/kg) 15 min before extubation and continued every 6 hours in the post-anesthesia care unit (PACU) afterward. At the end of the surgery, the infusion was stopped. Towards the end of the surgery, the gastric content of all patients was suctioned by an orogastric tube before extubation. All subjects were extubated awake in the operating room when able to open their eyes. All children were transferred to the post-anesthetic care unit (PACU). Standard monitoring was applied and they were observed for 2 hours. Liquids were given to the children in the PACU if they request. During their hospital stay in the oto-rhino-laryngology department, only soft diet was proposed to children. The intravenous infusion was kept until they reached an adequate oral intake.

**Data collection:** pre- and intraoperative data were collected including children's demographics, ASA score, heart rate, systolic, diastolic, and mean arterial pressure, and oxygen concentration in addition to surgery and anesthesia duration. Postoperative data were collected in the PACU by one of the investigators: frequency of POV, antiemetic consumption as well as the time of first oral intake in the oto-rhino-laryngology department, and before to be discharged from the hospital, the parent or the child (if age <8 years) reported: the number of vomiting episodes, the time of each episode, antiemetic consumption and the time to request the first oral intake. Only retching and vomiting episodes were recorded. Episodes of vomiting were considered as one episode if they occurred less than 5 min apart. Nausea is difficult to assess in children, so it was not recorded. If vomiting occurred more than twice; we treated with ondansetron 0.15 mg/kg.

**Variables:** our primary outcome was the presence of one episode of vomiting (an ejection of stomach contents through the mouth), retching (unproductive effort of vomiting), or both in the first 24 hours post operatively.

**Statistical analysis:** all analyses were performed with IBM SPSS®25.0.0 for windows. We tested normality of variables using the Shapiro-Wilk test. Results were reported as mean and standard deviation (SD), or frequency and percentage. We used analysis of variance (ANOVA) or the Kruskal-Wallis test for between-group comparisons, as appropriate. The X^2^ test and Fisher´s exact test were used for inferences on proportions. Data are expressed as mean standard deviation, median, minimum-maximum, or odds ratio 95% confidence interval (CI) unless otherwise stated. A two-sided P-value of 0.05 was considered significant.

**Ethical considerations:** we conducted this study after approval of the southern protection committee of people (C.P.P.SUD) under the aegis of the Health Ministry of the Tunisian republic, reference CPP SUD Nº 15/2019. Written and informed parental consent was obtained from all participants.

## Results

**Figure 1:**
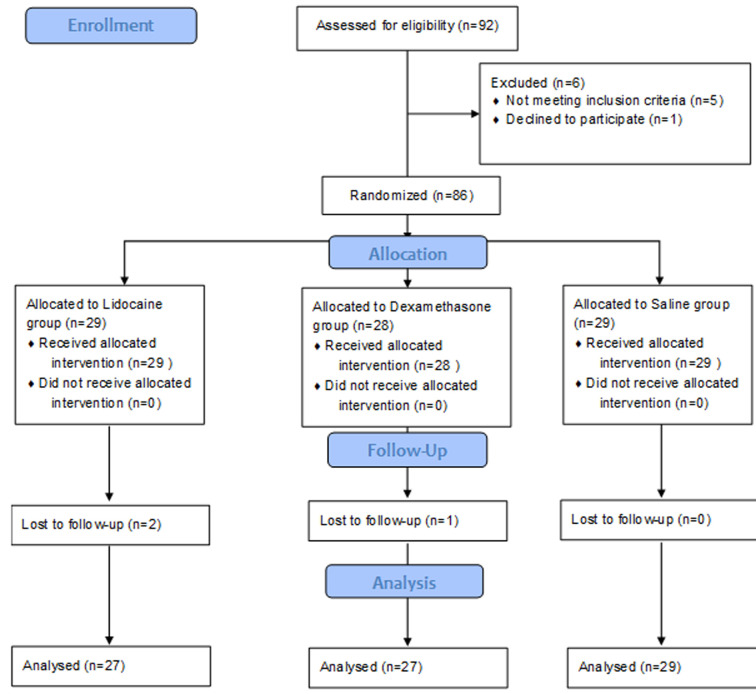
study flow diagram

We enrolled 86 children. One child randomized to the dexamethasone group and two children randomized to the lidocaine were excluded because of missing data. Finally, 83 children were analyzed and divided into 3 groups: 27 children for each lidocaine and dexamethasone group and 29 children for the Saline group (Figure 1). Our sample was characterized by a median of age =6 years with extreme (3-13), a sex ratio male/female =1.3, a median of weight =25 kg with extreme (12-55) and a height = 110 cm with extreme (80-160). The demographic data of the children and the duration of surgery and anesthesia were not significantly different between the three groups (Table 1). In the lidocaine group, 4 of 27 patients (14.8 %), experienced at least one episode of retching, vomiting, or both in the PACU, compared with 13 of 29 patients (44.8 %) in the saline group (p = 0.015, with an unadjusted OR of (0.214 95% CI, 0.059 to 0.777). In the Ward, one child from the lidocaine group (3.7%) experienced at least one episode of retching, vomiting, or both during the first 24 hour postoperative period, compared with 10 children from 29 (34.5%) in the saline group (p= 0.004, with an unadjusted OR of 0.073 95% CI: 009 to 0.621) (Table 2). In the dexamethasone group, 5 of 27 patients (18.5%), experienced at least one episode of retching, vomiting, or both in the PACU, compared with 13 of 29 patients (44.8 %) in the saline group (p= 0.035, with an unadjusted OR of 0.280 (95% CI, 0.083 to 0.944). In the ward, 3 children from the dexamethasone group (11.1%) experienced at least one episode of retching, vomiting, or both during the first 24 hour postoperative period, compared with 10 children from 29 (34.5%) in the saline group (p= 0.038, with an unadjusted OR of 0.238 95% CI, 0.057 à 0.986) (Table 2). Moreover, the difference between the dexamethasone group and the lidocaine group was non-significant in the PACU and the ward (Table 2). The time to the first oral intake was significantly shorter in the dexamethasone group and the lidocaine group compared with the saline group. However, there was no significant difference in the first oral intake between the Dexamethasone and the lidocaine group (Table 3).

**Table 1:** baseline and perioperative care data

	Saline group (n=29)	Dexamethasone group (n=27)	Lidocaine group (n=27)	Signification (p)
Boys/girls	14/15	18/9	15/12	0.378*
Age (year)	6 [3-13]	6 (3-13)	8 (4-12)	0.246⧧
Weight (kg)	25 (12-40)	25 (15-55)	28 (15-500	0.285⧧
Height (cm)	110 (80-135)	110 (84-150)	120 (82-160)	0.580⧧
Body mass index	20 (17-25)	20 (17-24)	20.83 (12-25)	0.233⧧
ASA Ⅰ/ASA Ⅱ	25/4	25/2	24/3	0.744*
Duration of surgery (min)	27.14 ± 8.3	26.11 ± 7.38	26.93 ± 9.53	0.893**
Duration of anesthesia (min)	37.07 ± 9.11	32.78 ± 8.69	36.48 ± 9.07	0.164**

Median (minimum-maximum); mean ± standard deviation; N: number of children; Kg: kilogram; cm: centimeter; min: minutes; p: the p-value between the 3 groups; ⧧: Kruskal-Wallis test; *: Pearson's Chi-squared test; **: ANOVA test.

**Table 2:** comparison of episodes of retching, vomiting, or both in the post-anesthetic care unit between study groups

	Saline group (n=29)	Dexamethasone group (n=27)	Lidocaine group (n=27)	Signification (p)
Episodes of retching, vomiting, or both in the PACU	44.8%	18.5%	14.8%	p1=0.035*
				p2=0.015*
				p3> 0.05⧧
Episodes of retching, vomiting, or both in the ward	34.5 %	11.1%	3.7%	p1=0.038*
				p2=0.004*
				p3 > 0.05⧧

N: number of children; p1: the p-value between the dexamethasone and control group; p2: the p-value between the lidocaine and control group; p3: the p-value between the lidocaine group and dexamethasone; Pearson's Chi-squared test; ⧧: Fisher's test

**Table 3:** comparison of the first oral intake between the 3 groups

	Saline group (n=29)	Dexamethasone group (n=27)	Lidocaine group (n=27)	Signification (p)	
First oral intake (h)	7 (4-9)	5 (4-6)	5 (4-6)	p=0.0001⧧	p1=0.0001*
					p2=0.0001*
					p3=0.900*

Median (minimum-maximum); N: number of children; h: hours; p: the p-value between the 3 groups; p1: the p-value between the dexamethasone and control group; p2: the p-value between the lidocaine and control group; p3: the p-value between the lidocaine group and dexamethasone; ⧧: Kruskal-Wallis test; *: Scheffe test.

## Discussion

We proposed to evaluate the efficacy of lidocaine and dexamethasone compared to saline infusion to reduce postoperative vomiting in children´s tonsillectomy. This double-blinded, randomized clinical trial shows that both infusion of lidocaine and dexamethasone given to children undergoing tonsillectomy under general anesthesia reduced POV compared with placebo treatment. Several studies focused on the effect of dexamethasone on POV in children tonsillectomy. They demonstrated that dexamethasone is effective in reducing POV during the first 24 hours post tensillectomy [9-12]. The recommendations of the Association of Pediatric Anesthetists of Great Britain and Ireland [13] concluded in 2016 that 0.15 mg/kg of dexamethasone provides a good reduction in POV without significant adverse effects in patients undergoing tonsillectomy. The mechanism of the anti-emetic effect of dexamethasone is still unknown; but its effectiveness is widely known and became indisputable [14]. In addition, corticosteroids have anti-inflammatory and analgesic effects, which improve recovery and accelerate the return to normal activity. Several studies suggest that the infusion of intravenous lidocaine during tonsillectomy could improve postoperative nausea and vomiting [3] a meta-analysis by Weibel *et al*. showed that an infusion of intravenous lidocaine reduced nausea 45/218 patients in the Lidocaine group vs. 66/222 in the control group, relative risk (RR) 0.82 (95% CI, 0.70 to 0.97), but didn´t improve vomiting between groups 0.49 (95% CI, 0.16 to 1.48) [15].

A systematic review and meta-analysis performed by Kranke *et al*. [16] suggested that lidocaines have an opioid-sparing effect, so it leads to fewer episodes of POV. Several studies have described the mechanisms of action for local anesthetics. Among these mechanisms; the binding of lidocaine to voltage-gated (Na+) channels have been described. This binding prevents the flow of Na+ ions through the channel pore [17]. Others mechanisms have been described, like the Blockade of muscarinic, nicotinie, and dopaminergic receptors, inhibition of opiate receptors and enhancement of gamma-aminobutyric acidinergic pathways. The anti-inflammatory properties of lidocaine have also been reported [15]. Another mechanism that may explain the anti-emetic properties of lidocaine is the inhibition the release of substance P, a potent NK1 agonist. So that, lidocaine may exert its anti-emetic effects through one or several of these mechanisms [18,19]. The beneficial effect of intravenous lidocaine on oral intake after tonsillectomy has not been studied. Studies have focused particularly on visceral surgery. In the controlled trial conducted by Kim *et al*. [20] the patients who received intravenous lidocaine administered for laparoscopic colorectal cancer surgery have a shorter time of oral intake after the surgery. Similar studies have been reviewed, they show that patients who were infused by dexamethasone during surgery have a faster first oral fluid intake, they also have an earlier and a better quality of oral intake. Aouad *et al*. [9] found that the first oral intake time in the dexamethasone group was 5.3 hours and group 10.9 hours in the placebo (p < 0.05) They also noted that the quality of oral intake was better at 24 hours postoperatively in the dexamethasone group. Elhakim *et al*. [10] showed that the first oral intake was faster in the dexamethasone group (0.4 ± 0.2 hours, compared with the placebo 1.3 ± 0.7 hours (p < 0.05)). They also noted that the quality of oral intake was higher than the control group. Pappas *et al*. [5] observed a better quality of oral intake in children post tonsillectomy who in dexamethasone group compared with those in a control group.

**Limitations:** the assessment of nausea in children is difficult. Despite the use of validated scales to evaluate nausea, symptoms were not very clear, mainly in young children. We did not measure lidocaine in the blood postoperatively because it is not a common practice in our institution, but we estimated that the doses injected are much lower than toxic doses; in addition, the children did not show any signs of local anesthetic toxicity.

## Conclusion

Intravenous lidocaine infusion is as effective and safe as a prophylactic single dose of dexamethasone for prophylaxis of postoperative nausea and vomiting and for improvement of oral intake after children’ tonsillectomy. The use of lidocaine reduce POV seems to be very attractive thinks to its efficacy, its low cost, and its availability as well as the absence of clinical toxicity.

### What is known about this topic

- *Postoperative vomiting (POV) is a frequent complication of tonsillectomy, with an incidence ranging from 23% to 73% without anti-emetic prophylaxis;*
- *Intravenous corticosteroids such as dexamethasone seems to be effective in reducing vomiting after tonsillectomy;*
- *Few studies are available for the infusion of lidocaine in children, the only study available doesn’t show benefit in patients undergoing tonsillectomies and was underpowered*.

### What this study adds

- *Both lidocaine infusion and dexamethasone given to children undergoing tonsillectomy under general anesthesia reduce the risk of postoperative vomiting compared with placebo treatment;*
- *Lidocaine may be infused safely in children;*
- *it's the only study which compares intravenous dexamethasone versus intravenous lidocaine in children*.
